# Supplementary material for: Identification of Driver Epistatic Gene Pairs Combining Germline and Somatic Mutations in Cancer
Source: Int J Mol Sci. 2023 May 26;24(11):9323. doi: 10.3390/ijms24119323 (PMC10253732; doi:10.3390/ijms24119323)
Supplement: Supplementary file 1 [file ijms-24-09323-s001.zip › supplementary-materials.pdf]

SUPPLEMENTARY MATERIALS

Identification of Driver Epistatic Gene Pairs Combining Germline and Somatic Mutations in Cancer

Jairo Rocha[1,2,*], *Jaume Sastre*[1], *Emilia Amengual-Cladera*[2,3], *Jessica Hernandez-Rodriguez*[2,3], *Victor Asensio-Landa*[2,3], *Damià Heine-Suñer*[2,3] *and Emidio Capriotti*[4]

[1] Department of Mathematics and Computer Science, University of the Balearic Islands, 07122 Palma de Majorca, Spain.
[2] Genomics of Health Group, Health Research Institute of the Balearic Islands (IDISBA), 07120 Palma de Majorca, Spain.
[3] Hospital Universitari Son Espases, 07120 Palma de Majorca, Spain.
[4] BioFolD Unit, Department of Pharmacy and Biotechnology (FaBiT), University of Bologna, 40126 Bologna, Italy.

Correspondence: jairo@uib.es

1 Colon Adenocarcinoma

We analyzed normal and matching tumor sample from 422 patients affected by colon adenocarcinoma (COAD). Our analysis starts with the detection of variants affecting protein sequence and the mutated gene in normal and tumor samples. For each possible gene pair, we calculated the contingency table and selected the gene pair with at least one Relevant Gene Pair Mutation (RGPM). For all the retained gene pairs, we calculated the epistatic and survival analysis p-values. In Table S1 we summarized the analysis of COAD samples in numeric terms.

1.1 Epistatic Gene Pairs and Survival Analysis

Starting for an initial set of ∼51 million gene pairs, we identify 450 of them with epistatic p-value $< 9.8\times10^{-10}$. All the contingency tables are reported in supplementary file 1. After this filter procedure, we calculated the Survival Analysis p-value, which is based on the comparison of the Overall Survival time of the patients with relevant gene-pair mutations (RGPMs) and those with background single gene mutations (BSGMs). Given the three possible mutation states: w (no mutation), b (germline) and s (somatic), RGPM correspond to gene pair mutations with the following states: (s, b), (b, s) and (s, s), while BSGMs comprises the states (w, b), (w, s), (b, w) and (s, w). Among the previously

identified gene pairs, only 16 of them have a Survival Analysis p-value lower than 0.05. In Table S2 we reported the contingency table of the 16 gene pair and in Figure S1 their corresponding Survival Analysis plots.

Table S1: Summary of the analysis of COAD samples in numeric terms.

COAD Samples	422
Variants Affecting Protein sequence	358,774
Mutated Genes	6,854
Gene Pairs	68,088,615
Gene Pairs with RGPMs	51,098,733
Epistatic Gene Pair (p-value $< 9.8 \times 10^{-10}$)	450
Epistatic Gene Pair with SA p-value < 0.05	16

Table S2: Contingency tables of the 16 epistatic gene pairs with p-value < 0.05. The mutation states of each gene (w, s and b) are defined in Materials and Methods. The relevant gene-pair mutations (RGPM) correspond to the contingency table elements (s, b), (b, s) and (s, s).

(A) CCDC73-HTR2B

g_1/g_2	w	s	b
w	359	1	18
s	7	8	2
b	27	0	0

(B) DDX4-KCNJ16

g_1/g_2	w	s	b
w	282	3	23
s	0	6	1
b	100	1	6

(C) DDX4-SNX13

g_1/g_2	w	s	b
w	287	16	5
s	0	7	0
b	102	1	4

(D) SEMG1-CYP2E1

g_1/g_2	w	s	b
w	217	0	19
s	3	6	1
b	147	1	28

(E) TRIP12-BTAF1

g_1/g_2	w	s	b
w	366	6	10
s	17	11	1
b	9	2	0

(F) ZNF99-HECTD2

g_1/g_2	w	s	b
w	191	0	7
s	1	4	3
b	206	5	5

(G) LGR5-MBD5

g_1/g_2	w	s	b
w	310	11	9
s	2	8	1
b	74	1	6

(H) TOPORS-FLT1

g_1/g_2	w	s	b
w	353	12	17
s	3	9	1
b	25	1	1

(I) ABCA8-C1orf168

g_1/g_2	w	s	b
w	248	0	26
s	18	8	1
b	89	3	29

(J) MAGI3-KIF20A

g_1/g_2	w	s	b
w	338	4	23
s	2	6	1
b	44	4	0

(K) RASAL2-TRIM37

g_1/g_2	w	s	b
w	366	9	22
s	6	9	1
b	8	0	1

(L) PHLPP1-CLASP1

g_1/g_2	w	s	b
w	351	11	18
s	8	10	1
b	20	0	3

(M) ZNF491-BTAF1

g_1/g_2	w	s	b
w	367	11	9
s	1	7	1
b	24	1	1

(N) GTF3C3-CATSPERB

g_1/g_2	w	s	b
w	285	7	42
s	2	7	3
b	63	1	12

(O) MTNR1B-VIT

g_1/g_2	w	s	b
w	292	5	49
s	4	7	0
b	48	1	16

(P) ARHGAP20-ZBTB39

g_1/g_2	w	s	b
w	342	2	31
s	8	7	3
b	26	1	2

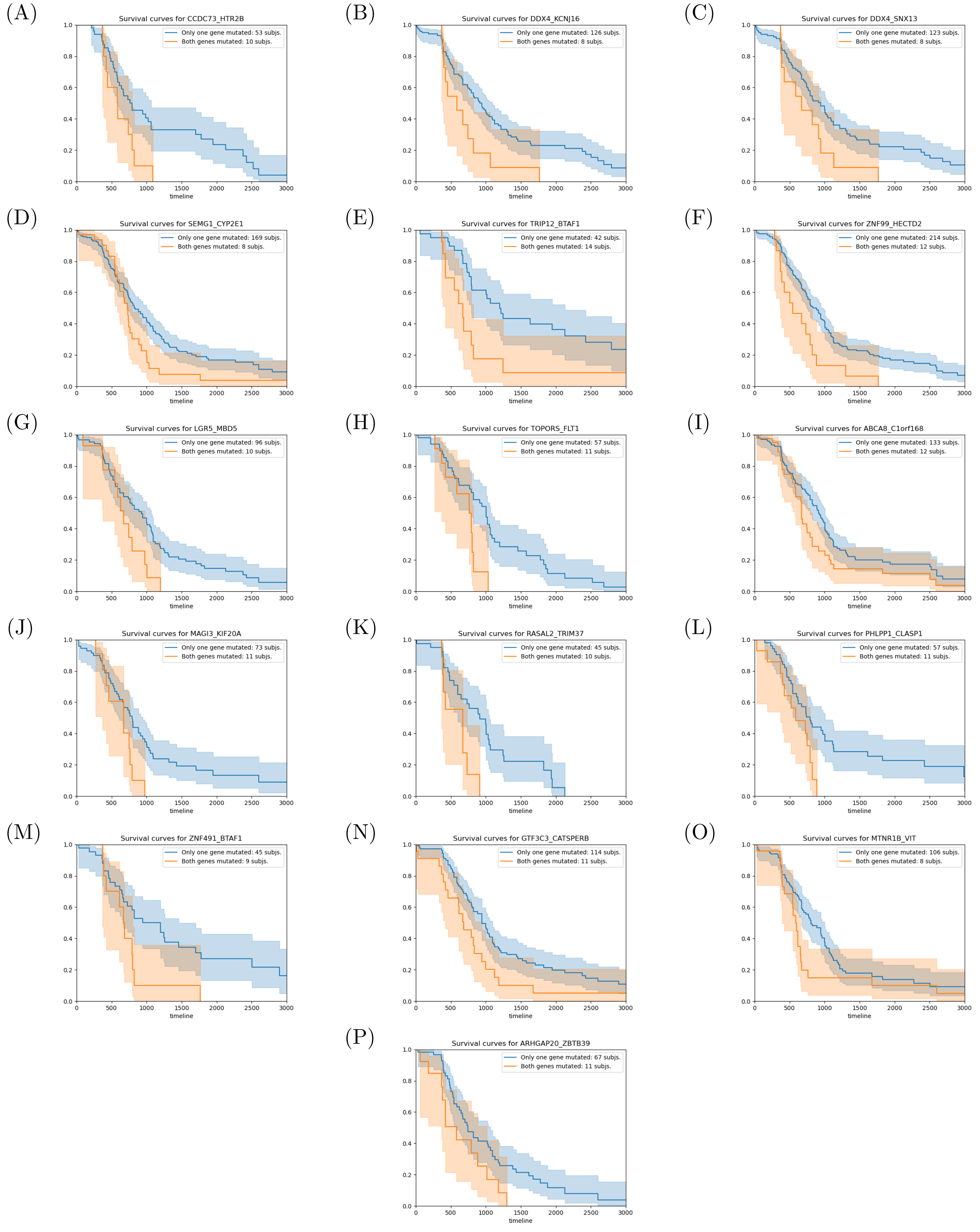

Figure S1: Survival analysis for 16 epistatic gene pairs with p-value < 0.05. The orange and blue curves represent the groups of subjects with RGPMs and BSGMs, respectively. RPGMs: (b, s), (s, b) and (s, s). BSGMs: (w, b), (w, s), (b, w) and (s, w). Mutation states are: w (no mutation), s (somatic) and b (germline).

2 Lung Adenocarcinoma

We analyzed normal and matching tumor sample from 405 patients affected by lung adenocarcinoma (LUAD). Our analysis starts with the detection of variants affecting protein sequence and the mutated gene in normal and tumor samples. For each possible gene pair, we calculated the contingency table and selected the gene pair with at least one Relevant Gene Pair Mutation (RGPM). For all the retained gene pairs, we calculated the epistatic and survival analysis p-values. In Table S3 we summarized the analysis of LUAD samples in numeric terms. Among the previously identified gene pairs, only 3 of them have a Survival Analysis p-value lower than 0.05. In Table S4 we reported the contingency table of the 3 gene pair and in Figure S2 their corresponding Survival Analysis plots.

Table S3: Summary of the analysis of LUAD samples in numeric terms

LUAD Samples	405
VAPs	192,304
Genes	6,854
Gene Pairs	23,485,231
Gene Pairs with RGPMs	10,961,208
Epistatic Gene Pair (p-value $< 5.0 \times 10^{-9}$)	4
Epistatic Gene Pair with SA p-value < 0.05	3

2.1 Epistatic Gene Pairs and Survival Analysis

Starting for an initial set of ∼10 million gene pairs, we identify 4 of them with epistatic p-value $< 5.0 \times 10^{-9}$. All the contingency tables are reported in supplementary file 1. After this filter procedure, we calculated the Survival Analysis p-value which is based on the comparison of the Overall Survival time of the patients with relevant gene-pair mutations (RGPMs) and those with background single gene mutations (BSGMs). Given the three possible mutation states: w (no mutation), b (germline) and s (somatic), RGPM correspond to gene pair mutations with the following states: (s, b), (b, s) and (s, s), while BSGMs comprises the states (w, b), (w, s), (b, w) and (s, w). Among the previously identified gene pairs, only 3 of them have a Survival Analysis p-value lower than 0.05. In Table 4 we reported the contingency table of the 3 gene pairs and in Figure 2 their corresponding Survival Analysis plots.

Table S4: Contingency tables of the CHRM2-SLC6A15 (A) and PSD2-SUGP1 (B) gene pairs. The mutation states of each gene (w, s and b) are defined in Materials and Methods. The relevant gene-pair mutations (RGPM) correspond to the contingency table elements (s, b), (b, s) and (s, s).

(A) CHRM2-SLC6A15

g_1/g_2	w	s	b
w	267	0	114
s	6	5	4
b	7	0	2

(B) PSD2-SUGP1

g_1/g_2	w	s	b
w	300	1	80
s	1	3	4
b	13	0	3

(C) UBR1-ACAD9

g_1/g_2	w	s	b
w	304	0	15
s	3	3	1
b	72	0	7

(A)

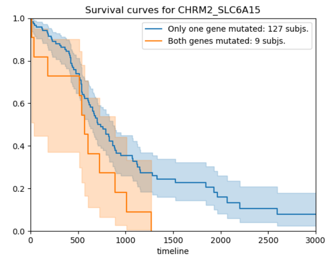

(B)

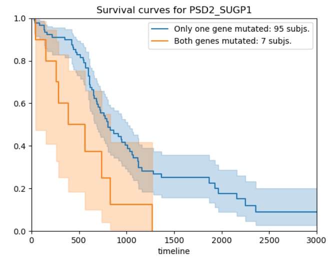

(C)

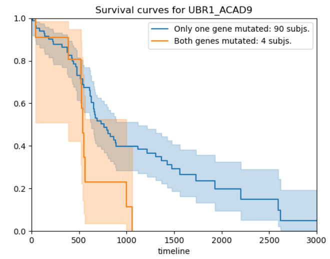

Figure S2: Survival analysis for the gene pairs CHRM2-SLC6A15 (A), PSD2-SUGP1 (B) and PSD2-SUGP1 (C). The orange and blue curves represent the groups of subjects with RGPMs and BSGMs, respectively. RPGMs: (b, s), (s, b) and (s, s). BSGMs: (w, b), (w, s), (b, w) and (s, w). Mutation states are: w (no mutation), s (somatic) and b (germline).
